# Carotid plaque characteristics and their association with cardiovascular risk factors and coronary atherosclerosis in a middle-aged population

**DOI:** 10.1016/j.jocmr.2026.102686

**Published:** 2026-01-08

**Authors:** Elin Good, Oscar Soto, Linda Bilos, Håkan Ahlström, Tamara Bianchessi, Jan Engvall, Isabel Gonçalves, My Troung, Ola Hjelmgren, David Marlevi, Bertil Wegmann, Petter Dyverfeldt

**Affiliations:** a Department of Cardiology in Linköping, and Department of Health, Medicine and Caring Sciences, Linköping University, Linköping, Sweden; b Center for Medical Image Science and Visualization (CMIV), Linköping University, Linköping, Sweden; c Division of Diagnostics and Specialist Medicine, Department of Health, Medicine and Caring Sciences, Linköping University, Linköping, Sweden; d Department of Thoracic and Vascular Surgery, Department of Health, Medicine and Caring Sciences, Unit of Cardiovascular Medicine, Linköping University, Linköping, Sweden; e Department of Surgical Sciences, Section of Radiology, Uppsala University, Uppsala, Sweden; f Antaros Medical, Mölndal, Sweden; g Department of Clinical Physiology and Department of Health, Medicine and Caring Sciences, Linköping University, Linköping, Sweden; h Department of Cardiology, Skåne University Hospital and Clinical Sciences Malmö, Lund University, Lund, Sweden; i Department of Diagnostic Radiology, Skåne University Hospital. Department of Clinical Sciences Lund, Lund University, Lund, Sweden; j Department of Molecular and Clinical Medicine Institute of Medicine, Sahlgrenska Academy, University of Gothenburg, Gothenburg Sweden; k Pediatric Heart Centre, Queen Silvia Children's Hospital, Sahlgrenska University Hospital, Region Västra Götaland, Gothenburg, Sweden; l Department of Molecular Medicine and Surgery, Karolinska Insititutet, Solna, Sweden; m Institute for Medical Engineering and Science, Massachusetts Institute of Technology, Cambridge, Massachusetts, USA; n Department of Computer and Information Science, Linköping University, Linköping, Sweden; o Science for Life Laboratory, Linköping University, Linköping, Sweden

**Keywords:** Carotid atherosclerosis, Lipid-rich necrotic core, Magnetic resonance angiography, Coronary artery disease, Cardiovascular risk factors

## Abstract

**Background:**

Carotid and coronary atherosclerosis are critical determinants of cardiovascular risk, yet their interrelationship in middle-aged populations is incompletely understood. This study assessed carotid plaque composition, risk-factor associations, coronary disease, and sex differences in a subclinical cohort.

**Methods:**

Within the Swedish CArdioPulmonary bioImage Study, 533 asymptomatic individuals aged 50–64 years with carotid plaque ≥2.7 mm on ultrasound underwent 3T multi-contrast carotid cardiovascular magnetic resonance (CMR) and coronary computed tomography angiography. Carotid plaque characteristics were determined manually using established criteria on multi-contrast weighted carotid CMR. Bayesian regression models evaluated associations between cardiovascular risk factors and coronary atherosclerosis.

**Results:**

Lipid-rich necrotic core (LRNC) was present in 60% (320/533) and intraplaque hemorrhage (IPH) in 5.4% (29/533); calcification occurred in 48.6% (259/533). Maximum carotid wall thickness was 1.8 (1.6-2.0) mm, and mean lumen area 31.3 (26.7-36.1) mm². Coronary atherosclerosis was present in 63.6% (339/533) of participants, with ≥50% stenosis in 12.9% (69/533), and coronary artery calcium score >400 in 12.8% (68/533). Men (N = 367) had larger carotid lumen area, mean wall area, and maximum wall thickness (all p < 0.001) than women (N = 166), differences that persisted after body-surface-area adjustment (all p < 0.01). LRNC was present in 66% (242/367) of men compared to 47% (78/166) of women (p < 0.001). LRNC presence was not associated with coronary atherosclerosis, whereas IPH was associated with coronary involvement.

**Conclusion:**

In middle-aged individuals, distinct cardiovascular risk factors were positively linked to the presence and volume of LRNC and calcified plaques. The substantial prevalence of high-risk plaque features, particularly LRNC and especially in men, highlights a significant subclinical carotid disease burden.

## Graphical abstract

**Figure:**
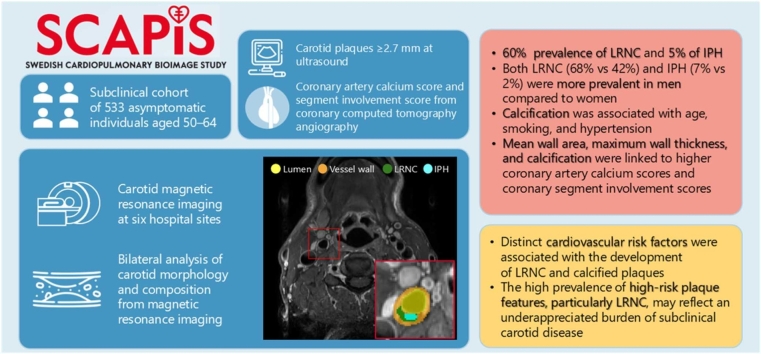
*LRNC* lipid-rich necrotic core, *IPH* intraplaque hemorrhage

## 1 Introduction

Carotid plaques are substrates for ischemic stroke and a hallmark of systemic atherosclerotic disease. Carotid plaques were detected in 61% of all middle-aged men and 49% of all middle-aged women in a recent population-based study, emphasizing the pervasive nature of this disease and its uneven distribution between men and women [1], [2]. Additionally, subclinical carotid plaques in this demography can progress substantially over only a few years [3]. These insights into the prevalence and potential progression of carotid plaques in middle-aged individuals highlight that early identification and personalized risk stratification of individuals with carotid atherosclerotic disease are paramount for reducing the burden of cardiovascular morbidity and mortality.

Clinical management of carotid plaques relies largely on geometric measures such as the degree of luminal stenosis. However, geometric measures insufficiently capture the complexity of plaque vulnerability. Substantial evidence supports the additional value of compositional features—particularly intraplaque hemorrhage (IPH) and lipid-rich necrotic core (LRNC)—which are associated with adverse plaque progression and elevated cardiovascular risk [4], [5]. Pathologically, LRNC represents a lipid and necrotic debris pool within the plaque, whereas IPH reflects extravasated erythrocytes and hemoglobin breakdown products within the lesion. The presence and volume of these high-risk plaque components can be measured with cardiovascular magnetic resonance (CMR) [6], [7], [8]. LRNC typically appears as a hypointense area on post-contrast T1-weighted images, and as iso-to-hypointense on native T1-weighted images and magnetization-prepared rapid acquisition gradient echo (MP-RAGE), whereas IPH appears hyperintense on native T1-weighted images and MP-RAGE and iso-to-hypointense on post-contrast T1-weighted images [8]. Despite the utility of carotid CMR in large-scale studies linking plaque characteristics to cardiovascular outcomes in older populations [7], [9], [10], comparable investigations of atherosclerotic plaque characteristics in middle-aged individuals are lacking, especially in the European setting.

Being a manifestation of systemic atherosclerosis, the assessment of carotid plaques may convey information relevant for other vascular beds. Specifically, the presence and characteristics of carotid plaques correlate with coronary artery disease [10], [11], [12]. For instance, carotid plaque burden has been linked to coronary artery calcium (CAC) levels [12], while LRNC has been associated with an increased risk of cardiovascular events [10]. These findings highlight the potential clinical value of carotid plaque characterization in cardiovascular risk assessment. However, the relationship between carotid characteristics and coronary atherosclerosis, as evaluated by coronary computed tomography (CT), remains underexplored—particularly in asymptomatic, middle-aged populations.

The Swedish CardioPulmonary bioImage Study (SCAPIS) uniquely addresses an important gap in population-based studies of carotid plaque characteristics by coupling carotid CMR with comprehensive cardiovascular phenotyping, including coronary CT (angiography and calcium scoring). Within SCAPIS, 607 individuals with ultrasound-detected carotid plaque ≥2.7 mm underwent carotid CMR, enabling integrated assessment of carotid and coronary atherosclerosis in middle-aged individuals.

The aim of this study was to quantify carotid plaque characteristics and their relationship to cardiovascular risk factors and coronary plaque characteristics in this asymptomatic, population-based cohort, including differences between women and men.

## 2 Methods

### 2.1 Study participants

SCAPIS is a national study in which 30,154 individuals aged 50-64 years were examined between 2012 and 2018. Participants underwent extensive imaging—including coronary CT—together with pulmonary and cardiovascular functional testing, blood sampling (glucose, lipids, and other relevant biomarkers), and a detailed questionnaire on medical history, lifestyle, and socioeconomic status [13]. SCAPIS was approved by the Umeå Ethical Review Board (registration number 2010-228-31M), and all participants provided written informed consent. The present add-on CMR study was additionally approved by the Ethical Review Authority of Sweden (registration number 2022-04459-01).

Data on coronary artery disease include CAC score and segment involvement score (SIS). CAC scoring was made on non-contrast CT, as described previously [14]. SIS was determined based on the 18 coronary segment model defined by the Society of Cardiovascular Computed Tomography using coronary computed tomography angiography (CorCTA) [15]. To improve reading and ensure focus on the most clinically significant findings, the analysis prioritized 11 key coronary segments (segments 1-3, 5-7, 9, 11-13, and 17), which were mandatory to report. The remaining segments were documented only if they demonstrated evidence of atherosclerosis or calcium blooming.

In SCAPIS, all subjects underwent carotid ultrasound measurements of maximum plaque height measured from the adventitia–media interface to the luminal surface at the carotid bifurcation (common or internal carotid artery), bilaterally. Participants in the current study were recruited among individuals in SCAPIS who had at least one carotid plaque ≥2.7 mm at ultrasound. This inclusion criterion was adapted from prior studies using ≥2.5 mm [16] and calibrated in a SCAPIS pilot to yield ∼6-7% eligibility.

### 2.2 Carotid plaque characterization with CMR

**Fig. 1:**
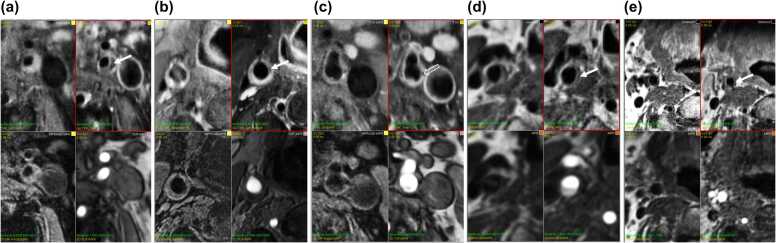
Example images in (a) a 61-year-old male acquired at site 1 with a dedicated carotid coil, (b) a 59-year-old female acquired at site 2 with a dedicated carotid coil, (c) a 55-year-old male acquired at site 1 with a dedicated carotid coil, (d) a 57-year-old male acquired at site 4 with a standard neck coil, and (e) a 57-year-old female acquired at site 4 with a standard neck coil. All images are axial and oriented perpendicular to the carotid artery. For each subject, the four carotid images are native T1-weighted (upper left), post-contrast T1-weighted (upper right), MP-RAGE (lower left), and TOF (lower right). Solid arrow: lipid-rich necrotic core. Open arrow: calcification. All scans were performed at 3T. Sites 1 and 4 used gadobutrol (Gadovist, Bayer Healthcare, Leverkusen, Germany), and site 2 used gadoterate (Dotarem, Guerbet, Villepinte, France), each administered at 0.2 mmol/kg body weight. Full imaging parameters, including post‑contrast timing and imaging planes, are detailed in Supplemental Table S1. *MP-RAGE* magnetization-prepared rapid acquisition gradient echo, *TOF* time-of-flight

A multi-contrast-weighted carotid CMR protocol, consisting of a localizer followed by four bilateral high-resolution acquisitions centered at the carotid bifurcation, was used at all five sites. The number of datasets per site was 315, 180, 60, 35, and 17 for sites 1-5, respectively. The protocol consisted of a stack of T1-weighted images before and after administration of a gadolinium-based contrast agent (Supplemental Table S1); three-dimensional (3D) magnetization-prepared rapid gradient-echo (MP-RAGE); and 3D time-of-flight magnetic resonance (MR) angiography (Fig. 1). All MR scans were obtained using 3T MR scanners. Scanner manufacturers, coil equipment, and sequence parameters varied across sites (Supplemental Table S1). A total of 84% (510/607) of the data were acquired using a 3T MR scanner (Achieva, Philips Healthcare, Best, the Netherlands) in combination with an 8-channel dedicated carotid coil (Shanghai Chenguang Medical Technologies, Shanghai, China). The remaining 16% (97/607) were acquired using a 3T Siemens Healthineers MR scanners (Skyra and Aera) using a standard neck coil (Supplemental Table S1). Representative images acquired with the dedicated carotid coil and the standard neck coil are shown in Fig. 1. Inter-coil quality assurance was not performed.

Bilateral analysis of carotid morphology and composition in the multi-contrast-weighted carotid MR images was conducted using Vessel Mass (Medis Medical Imaging, Leiden, the Netherlands). The analysis included manual segmentation of the lumen and vessel wall followed by manual delineation of carotid plaque composition performed by an observer with 6 months of dedicated training on carotid MR image analysis. Carotid plaque characteristics in terms of geometric variables and LRNC and IPH (termed high-risk plaque components) as well as calcification were assessed both for the left and right carotid arteries. Calcification was identified as a hypointense area in all images; LRNC, as a hypointense area in post-contrast T1-weighted images, and as iso- or hypointense area in MP-RAGE images; IPH, as hyperintense area in MP-RAGE images or pre-contrast T1-weighted images if MP-RAGE was nondiagnostic; and ulcer, as hyperintense area in time-of-flight image, and as hypointense area in the other images [8]. For plaque components, both presence and volume were measured, with no minimum size threshold.

Carotid artery morphology was measured as the average vessel area, average wall area, maximum wall thickness, and the normalized wall index defined as (total vessel area − lumen area)/total vessel area [17]. Additionally, each subject was categorized according to carotid Carotid Plaque Reporting and Data System (Plaque-RADS) based on plaque height and presence of LRNC and IPH, ranging from Plaque-RADS I (absence of plaque) to Plaque-RADS IV (complicated plaque) [18].

### 2.3 Statistical analysis

Preliminary analyses were performed in SPSS Statistics 28 (IBM Corp. Armonk, New York,). Continuous variables were tested for normality using the Shapiro-Wilk test. Variables that did not follow a normal distribution were reported as medians with interquartile ranges (IQR). For normally distributed variables, means with standard deviations (SD) were reported. Non-parametric statistical methods were employed for comparisons of non-normally distributed data, while parametric methods were used for normally distributed data where applicable. Group comparisons for men vs women were performed using the chi-square test for categorical variables and Student's t-test for continuous variables. A p-value <0.05 was considered significant. Power analysis to investigate the total sample size needed for sex-difference analyses was not prespecified. Post hoc, for LRNC presence, the study had >99% power at α = 0.05 (two-sided) to detect the observed men–women difference (66% [n = 242/367] vs 47% [n = 78/166]). An intra-observer variability study was done to evaluate the repeatability of the identification of plaque components. The prevalence-adjusted bias-adjusted Cohen's kappa, with a 95% confidence interval, was computed based on repeated measurements in 100 carotid bifurcations after a washout period of >2 years. Data for the intra-observer variability study were randomly selected from the 533 subjects. An inter-observer variability study was done in 13% (69/533) of the data to evaluate the reproducibility of lumen area and total vessel area; the interclass correlation coefficient was 97% for the lumen contour, and 93% for the outer wall contour.

The main analyses were performed in R (R Foundation for Statistical Computing, Vienna, Austria) using Bayesian zero-inflated negative binomial (ZINB) regression to predict a response variable for coronary atherosclerosis using measures of carotid morphology and composition as predictors. This type of model suits our data as our response variables are generally inflated by many zeros. The Bayesian ZINB regression was implemented by using default, weakly informative priors [19]. Each measure of carotid composition was modeled by a log-normal hurdle model, as the values of each measure are either zero or a continuous measurement above zero. The log-normal distribution was chosen for the continuous part due to its ability to handle outliers. The hurdle model consists of a binary part of values equal to zero or not, which was analyzed by Bayesian logistic regression using the default non-informative prior distributions in R package UPG [20], and a continuous part of values above zero, which was analyzed by Bayesian linear regression for the log of the values using non-informative prior distributions. Associations between the outcome variable and a predictor were judged as strong if the 95% posterior interval for the corresponding parameter of the predictor did not include zero. Given the expected low IPH prevalence and small volumes in this middle-aged asymptomatic cohort, we used IPH presence in all inferential analyses and reported IPH volume descriptively only.

## 3 Results

### 3.1 Study population: Cohort characteristics, cardiovascular risk factors, and coronary artery disease

Of 2462 eligible participants with ≥2.7 mm carotid plaque, 607 underwent carotid CMR; 74 were excluded due to missing data in one or more image series, yielding 533 participants with complete CMR (age 59 ± 4.3 years; 69% (367/533) men). Participation varied across sites due to scheduling and logistical constraints in this optional substudy, which limited throughput at some centers. Among the 533, 68.5% (365/533) were overweight (body mass index [BMI] was 27 ± 3.7 kg/m²), ∼20% (103/533) were active smokers. Furthermore, approximately one-third of the participants reported a history of hypertension, 11% (61/533) were on lipid-lowering therapy, 5% (27/533) were treated for diabetes mellitus, and 2.5% (13/533) used insulin. Baseline characteristics for the CMR group, all SCAPIS participants with carotid plaques ≥2.7 mm, and the full SCAPIS cohort are detailed in Table 1. As expected, the CMR group had higher risk factor levels than the overall SCAPIS population, similar to the pattern seen for the ≥2.7 mm group.

**Table 1:** Characteristics of SCAPIS participants with and without carotid CMR.

Characteristic	All carotid CMR			All with plaque ≥2.7 mm	All SCAPIS participants	Differences men/women	Differences CMR/≥2.7 mm	Differences CMR/all
		Carotid CMR Men	Carotid CMR Women			p	p	p
Sample size—n	533	367	166	2462	27,647			
*Sociodemographics*								
Age (y)	60 (56-63)	60 (56-63)	60 (55-63)	57 (54-61)	57 (54-61)	0.820	<0.05	<0.001
Education, university degree—n (%)	205 (38.5)	134 (36.5)	71 (42.8)	739 (38.1)	12,183 (44.1)	0.433	0.054	0.088
*Anthropometry*								
Body mass index (kg/m^2^)	26.6 (24.6-29.3)	26.8 (24.9-28.9)	26.4 (23.2-31.1)	27.1 (24.7-29.9)	26.3 (23.9-29.4)	0.411	<0.05	0.077
Waist circumference (cm)	96.0±11.4	98.2±9.8	91.0±13.1	98.0±12.5	94.4±12.9	<0.001	<0.001	<0.05
*Behavior*								
Smoking status								
Current smoker—n (%)	103 (19.3)	69 (18.8)	34 (20.5)	440 (22.0)	3638 (13.2)	0.710	0.275	<0.001
Former smoker—n (%)	201 (37.7)	124 (33.8)	77 (46.4)	779 (39.0)	9818 (35.5)	0.066	0.719	0.463
Cigarette pack years	14.7 (6.0-26.8)	13.5 (5.0-27.6)	16.0 (7.3-24.5)	18 (8.3-31.5)	12.9 (5.5-24)	0.704	<0.001	0.059
Duration of smoking (y)	30 (14-40)	28 (12-41)	31 (19-40)	33 (18-42)	25 (13-37)	0.462	<0.05	<0.05
Alcohol use								
Once per month or less—n (%)	70 (13.1)	43 (12.0)	27 (16.4)	294 (15.3)	4091 (14.8)	0.243	0.828	0.321
2-4 times per month—n (%)	205 (38.5)	134 (37.5)	71 (43.0)	623 (32.3)	9996 (36.2)	0.433	<0.05	0.492
More than once a week—n (%)	37 (6.9)	31 (8.7)	6 (3.6)	200 (10.4)	2044 (7.4)	<0.05	<0.05	0.683
Physical activity during leisure time								
None, or physically active <2 hrs/week—n (%)	55 (10.3)	39 (11.2)	16 (9.8)	274 (14.3)	3169 (11.5)	0.664	0.626	0.649
Moderate, physically active >4 hrs/week—n (%)	246 (46.2)	157 (45.2)	89 (54.6)	905 (47.2)	12,290 (44.5)	0.249	1.000	0.627
Moderate but regular, including high-intensity—n (%)	150 (28.1)	44 (27.0)	150 (29.4)	481 (25.1)	7738 (28.0)	0.542	0.183	0.667
Regular exercise, including high-intensity—n (%)	59 (11.1)	45 (13.0)	14 (8.6)	193 (10.1)	3034 (3.2)	0.196	0.440	0.819
*Treatment*								
Cholesterol-lowering medication—(%)	61 (11.4)	48 (13.1)	13 (7.8)	295 (14.8)	2052 (7.4)	0.078	0.050	<0.001
Antihypertensive medication—n (%)	155 (29.1)	114 (31.1)	41 (24.7)	659 (33.0)	5186 (18.8)	0.134	0.087	<0.001
Diabetes medication—n (%)	27 (5.1)	18 (4.9)	9 (5.4)	167 (8.4)	988 (3.6)	0.801	<0.05	0.061
Blood pressure								
Systolic blood pressure (mmHg)	134 (122-146)	134 (123-144)	134 (121-148)	130 (120-143)	124 (114-136)	0.860	<0.05	<0.001
Diastolic blood pressure (mmHg)	80 (73-88)	81 (73-88)	80 (73-90)	79 (72-86)	77 (70-84)	0.447	<0.001	<0.001
*Clinical chemistry*								
Total cholesterol (mmol/L)	5.6±1.1	5.4±1.0	5.8±1.1	5.5±1.2	5.5±1.1	<0.001	0.737	0.771
HDL cholesterol (mmol/L)	1.4 (1.2-1.8)	1.4 (1.1-1.6)	1.4 (1.4-2.1)	1.4 (1.2-1.8)	1.6 (1.3-1.9)	<0.001	0.104	<0.001
LDL cholesterol (mmol/L)	3.5 ±1.0	3.5±1.0	3.5±1.1	3.6±1.0	3.5±1.0	0.866	0.284	0.086
Triglycerides (mmol/L)	1.2 (0.8-1.4)	1.2 (0.8-1.8)	1.1 (0.8-1.4)	1.2 (0.9-1.7)	1.0 (0.8-1.5)	0.113	0.252	<0.001
HbA1c (mmol/mol)	36 (33-39)	36 (33-38)	36 (34-39)	36 (34-39)	35 (33-38)	0.305	<0.05	<0.001
High-sensitivity C-reactive protein (mg/L)	1.1 (0.6-2.1)	1.0 (0.6-1.9)	1.4 (0.7-3.0)	1.2 (0.6-2.6)	1.0 (0.6-2.2)	<0.001	0.114	<0.001
eGFR (mL/min/1.73 m^2^)	75.4±9.5	74.6±9.5	77.1±9.5	76.2±10.5	77.1±9.8	<0.05	<0.05	0.510
*Risk scores*								
SCORE (%)	1.8 (1.0-2.9)	2.3 (1.5-3.5)	0.9 (0.5-1.5)	1.7 (1.0-2.9)	1.0 (0.6-1.8)	<0.001	0.411	<0.001
*Prevalent cardiovascular disease*								
Stroke—n (%)	15 (2.8)	10 (2.7)	5 (3.0)	62 (3.1)	494 (1.8)	0.853	0.630	0.166
Myocardial infarction—n (%)	22 (4.1)	17 (4.6)	5 (3.0)	104 (5.2)	503 (1.8)	0.384	0.397	<0.001
Heart failure—n (%)	4 (0.8)	3 (0.8)	4 (0.8)	29 (1.5)	190 (0.7)	0.790	0.299	0.833
COPD—n (%)	8 (1.5)	7 (1.9)	8 (1.5)	91 (4.6)	525 (1.9)	0.251	<0.05	0.671

*SCAPIS* Swedish CArdioPulmonary bioImage Study, *HDL* high-density lipoprotein, *IQR* interquartile range, *LDL* low-density protein, *eGFR* estimated glomerular filtration rate, *SCORE* Systematic COronary Risk Evaluation, *COPD* chronic obstructive pulmonary disease, *CMR* cardiovascular magnetic resonance, *SD* standard deviations

Data were assessed for normality using the Shapiro-Wilk test. Variables that did not meet the assumption of normality were summarized using medians and interquartile ranges (IQR), while normally distributed variables were summarized using means and standard deviations

P-values (p) were calculated to assess differences between groups. A chi-square test was used for categorical variables, while Student's t-test was applied for continuous variables with a normal distribution. For non-normally distributed continuous variables, the Mann-Whitney U test was used. Continuous variables are presented as mean ± SD for normally distributed data or as median (IQR) for non-normally distributed data

Despite the low inclusion in the CMR study at some sites, the N = 533 CMR group and the whole N = 2463 ≥2.7 mm plaque group were broadly similar. The CMR group was older with higher systolic and diastolic blood pressure, but had lower BMI and waist circumference and reported lower rates of smoking and alcohol consumption, suggesting no major discrepancies in background profiles. Similarly, when assessing coronary characteristics, the CMR group displayed more advanced disease patterns in certain coronary territories, while in others, disease burden was lower, with comparable calcium scores between groups—indicating overall similarities in cardiovascular profiles (Table 2).

**Table 2:** Coronary atherosclerosis in SCAPIS participants with and without carotid CMR.

Coronary characteristics—n (%)	All carotid CMR	Carotid CMR Men	Carotid CMR Women	All with plaque ≥2.7 mm	All SCAPIS participants	Differences men/women	Differences CMR/≥2.7 mm	Differences CMR/all
						p	p	p
Sample size	533	367	166	2462	27,647			
Atherosclerosis in any coronary segment	339 (63.6)	258 (73.1)	81 (50.3)	1248 (62.5)	10,728 (38.8)	<0.001	<0.001	<0.001
Any stenosis ≥50%	69 (12.9)	58 (16.4)	11 (6.8)	358 (14.5)	1410 (5.1)	<0.001	<0.001	<0.001
Left main disease (stenosis ≥50%)	4 (0.8)	3 (0.8)	1 (0.6)	22 (0.9)	53 (0.2)	-	<0.001	<0.01
Proximal LAD disease (stenosis ≥50%)	34 (6.4)	27 (7.6)	7 (4.3)	135 (5.5)	512 (1.9)	<0.001	<0.001	<0.001
LCX disease (stenosis ≥50%)	19 (3.6)	17 (4.8)	2 (1.2)	96 (3.9)	267 (1.0)	<0.001	<0.001	<0.001
RCA disease (stenosis ≥50%)	17 (3.2)	15 (4.2)	2 (1.2)	113(4.6)	375 (1.4)	<0.001	<0.001	<0.001
Only noncalcified plaques	20 (3.8)	15 (4.2)	20 (3.9)	51 (2.1)	627 (2.3)	<0.001	<0.05	<0.001
Any plaque noncalcified	48 (9.0)	41 (11.2)	48 (9.3)	308 (12.5)	1522 (5.5)	<0.001	<0.05	<0.001
*Calcium score—n (%)*								
0	179 (35.0)	98 (26.7)	81 (48.8)	746 (30.3)	10,792 (39.0)	<0.001	0.099	<0.001
>0	332 (65.0)	269 (73.3)	85 (51.2)	1401 (70.1)	15,712 (56.8)	<0.001	0.099	<0.001
1-10 (ultralow)	61 (11.4)	40 (10.9)	21 (12.7)	265 (10.8)	3031 (11.0)	<0.001	0.558	0.719
11-100 (low)	109 (20.5)	82 (22.3)	27 (16.3)	545 (22.1)	4537 (16.4)	<0.001	0.306	<0.05
101-400 (moderate)	94 (17.6)	69 (18.8)	25 (15.1)	422 (17.1)	2196 (7.9)	<0.001	0.675	<0.001
>400 (high)	68 (12.8)	60 (16.3)	8 (4.8)	333 (13.5)	1028 (3.7)	<0.001	0.566	<0.001
CACS—median (IQR)	15 (0-150)	34 (0-226)	0.5 (0-73)	23 (0-174)	0 (0-20)	<0.001	0.098	<0.001
*Segment Involvement Score (SIS)*								
SIS—median (IQR)	1 (0-4)	2 (0-5)	1 (0-2)	2 (0-4)	0 (0-2)	<0.001	<0.05	<0.001

*CMR* cardiovascular magnetic resonance*, LAD* left anterior descending artery*, LCX* left circumflex artery*, RCA* right coronary artery*, CACS* coronary artery calcium score*, SIS* Segment Involvement Score, *IQR* interquartile ranges, *SCAPIS* Swedish CArdioPulmonary bioImage Study

Each p-value (p) is based on a test for differences between groups, where a chi-square test and a Mann-Whitney U test were used for each categorical and continuous variable, respectively. For left main disease, the number of participants with ≥50% stenosis was too low for statistical analysis comparing men and women in the CMR group

Coronary artery disease was more prevalent and advanced in the study population that underwent carotid CMR than in the overall SCAPIS cohort, emphasizing the systemic nature of atherosclerosis (Table 2). CorCTA-detected atherosclerosis was observed in 63.6% of the study population, with significant stenosis (>50%) in 12.9%, left main, proximal left anterior descending, or three-vessel disease in 7.1%, and noncalcified plaques in 9.0% of the study population.

### 3.2 Carotid plaque characteristics

LRNC and IPH were identified in 60% (320/533) and 5% (29/533) of the participants, respectively (Table 3). No significant differences in LRNC or IPH prevalence were observed between the youngest 1/3 and the oldest 1/3 of the study cohort. Carotid calcification was observed in 49% (259/533) of the participants. Median LRNC volume was 18 (0-55) mm^3^ and maximum wall thickness 1.8 (1.6-2.0) mm (Table 3). The majority of carotid plaques in this cohort were categorized as plaque-RADS II (N = 502), corresponding to moderately vulnerable plaques. A smaller subset of individuals fell into higher risk categories, with two individuals classified as plaque-RADS III and 29 as plaque-RADS IV. The prevalence-adjusted bias-adjusted Cohen's kappa, with a 95% confidence interval, was = 0.96 [0.86, 1.00], 0.76 [0.60, 0.87], and 0.92 [0.80, 0.98] for IPH, LRNC, and calcification, respectively.

**Table 3:** Carotid plaque characteristics, by age category and sex.

Characteristics		Total	Men			Women			All men	All women	p	Adj p
			50-54 y	55-59 y	60-65 y	50-54 y	55-59 y	60-65 y				
Sample size		533	87	101	179	52	36	78	367	166	533	533
Calcification, presence	n (%)	259 (48.6)	37 (42.5)	47 (46.5)	100 (55.9)	18 (34.6)	16 (44.4)	41 (52.6)	184 (50.1)	75 (45.2)	0.167	0.099
Calcification, volume (mm^3^)	median (IQR) Mean±SD	0 (0-15.2) 12.7±2.4	0 (0-9.7) 10.1± 21.3	0 (0-16.0) 12.0±21.5	0 (0-21.0) 15.6±26.7	0 (0-9.5) 9.0±19.5	0 (0-9.4) 7.7±20.2	4.8 (0-14.8) 14.4±25.8	0 (0-17.4) 13.3±24.0	0 (0-11.0) 11.2±22.9	0.176	0.178
IPH, presence	n (%)	29 (5.4)	6 (6.9)	7 (6.9)	12 (6.7)	2 (3.8)	0 (0)	2 (2.6)	25 (6.8)	4 (2.4)	<0.05	0.223
IPH, volume (mm^3^)—mean±SD	Median (IQR) Mean±SD	0 (0-0) 2.5±13.4	0 (0-0) 2.2 ±10.1	0 (0-0) 2.9±13.5	0 (0-0) 4.2±18.8	0 (0-0) 0.5±2.9	0 (0-0) 0±0	0 (0-0) 1.2±7.7	0 (0-0) 3.4±15.7	0 (0-0) 0.7±5.6	<0.05	0.121
LRNC, presence	n (%)	320 (60.0)	57 (65.5)	62 (61.4)	123 (68.7)	29 (55.8)	12 (33.3)	37 (47.4)	242 (65.9)	78 (47.0)	<0.001	<0.01
LRNC, volume (mm^3^)—mean±SD	Median (IQR) Mean±SD	17.6 (0-55.3) 43.4±71.2	30.4 (0-74.6) 48.7±61.8	21.6 (0-54.0) 48.5±79.4	21.3 (0-67.0) 52.7±82.4	13.3 (0-53.2) 32.8±51.8	0 (0-23.2) 15.7±30.0	0 (0-34.0) 29.4±62.5	24.1 (0-65.6) 50.6±77.0	0 (0-34.9) 27.5±53.7	<0.001	<0.05
Maximum wall thickness (mm)—mean±SD	Median (IQR) Mean±SD	1.8 (1.6-2.0) 1.8±0.3	1.8 (1.6-2.0) 1.8±0.3	1.8 (1.6-2.0) 1.9±0.3	1.9 (1.7-2.1) 1.9±0.3	1.7 (1.6-1.9) 1.8±0.3	1.6 (1.5-1.8) 1.6±0.3	1.8 (1.5-1.9) 1.8±0.3	1.9 (1.7-2.1) 1.9±0.3	1.7 (1.5-1.8) 1.7±0.3	<0.001	<0.01
Fibrous cap area (mm^2^)—mean±SD	Median (IQR) Mean±SD	1.6 (0-3.0) 1.9±2.0	2.1 (0-3.5) 2.2±2.1	1.6 (0-3.3) 2.1±2.2	1.9 (0-3.6) 2.3±2.1	1.4 0-2.6) 1.5±1.6	0 (0-1.9) 0.9±1.4	0 (0-2.2) 1.2±1.7	1.9 (0-3.5) 2.2±2.1	0 (0-2.2) 1.2±1.6	<0.001	<0.001
Normalized wall index—mean±SD	Median (IQR) Mean±SD	0.51 (0.48-0.54) 0.51±0.04	0.50 (0.47-0.53) 0.50±0.04	0.51 (0.48-0.55) 0.51±0.04	0.51 (0.49-0.54) 0.52±0.04	0.51 (0.49-0.54) 0.52±0.04	0.51 (0.48-0.54) 0.51±0.04	0.53 (0.49-0.56) 0.53±0.04	0.51 (0.48-0.54) 0.51±0.04	0.52 (0.49-0.55) 0.52±0.04	<0.05	0.065
Wall area (mm^2^)—mean±SD	Median (IQR) Mean± SD	32.9 (28.7-38.1) 33.9±7.0	33.5 (28.8-36.9) 33.8±6.0	34.0 (30.6-38.8) 34.9±6.5	36.2 (31.3-41.1) 36.9±7.7	29.6 (26.3-32.9) 30.0±5.0	27.8 (25.4-31.8) 28.7±5.2	30.8 (26.1-33.8) 30.9±5.0	34.5 (30.5-39.9) 35.6±7.1	29.9 (26.4-32.9) 30.1±5.1	<0.001	<0.001
Lumen area (mm^2^)—mean±SD	Median (IQR) Mean±SD	31.3 (26.7-36.1) 31.9±7.6	33.1 (29.3-36.9) 33.7±7.1	33.3 (28.3-37.6) 33.3±7.2	32.4 (29.1-38.2) 34.3±7.9	27.0 (24.0-31.8) 27.7±5.7	26.5 (23.5-31.6) 27.7±5.5	26.9 (22.8-32.4) 27.7±6.2	32.5 (28.9-37.8) 33.9±7.5	26.9 (23.5-31.8) 27.7±5.9	<0.001	<0.001

*IPH* intraplaque hemorrhage, *LRNC* lipid-rich necrotic core, *CACS* coronary artery calcium score, *CMR* cardiovascular magnetic resonance, *SD* standard deviations, *IQR* interquartile ranges.

Plaque characteristics were assessed using CMR. Continuous variables are non-normally distributed, both median and mean values are presented to facilitate comparisons. The presence of zeros in a majority of participants for some variables skews the distribution, which should be considered when interpreting the data. Each p-value (p) represents the statistical test for differences between sexes. For categorical variables, a chi-square test was used. For continuous variables, differences were assessed using the Mann-Whitney U test. In the adjusted model, body surface area (BSA) was included as a covariate: continuous variables were analyzed using a general linear regression model, while categorical variables were analyzed using binary logistic regression. Volume measurements represent the total bilateral values, including both the left and right carotid arteries. Ulcer presence and volume are not reported in the table, as only two participants had plaques with these characteristics. The area measurements are presented as the mean area of a single vessel, while maximum wall thickness reflects the mean of the highest recorded values

The statistical analysis based on ZINB modeling revealed several significant positive relations between carotid plaque morphology and carotid plaque composition. Increased maximum wall thickness, normalized wall index, mean wall area, and mean lumen area were, respectively, strongly associated with the presence and volume of LRNC (Table 4, Supplemental Fig. S2). Calcification was positively associated with the presence and volume of maximum wall thickness, normalized wall index, and mean wall area (Table 4). The effect size for maximum wall thickness was larger, on average, compared to the other predictors. Figures of these associations are shown in Supplemental Fig. S2.

**Table 4:** Plaque morphology for the prediction of plaque calcification and lipid-rich necrotic core (LRNC).

	Posterior mean	Posterior SD	2.50%	97.50%	Prob pos eff
Y = Calcification presence (volume > 0). Log(y) = X*β + ε (simple linear regressions)^1^					
Intercept	−4.13	0.07	−4.26	−4	100
Maximum wall thickness	0.23	0.07	0.1	0.36	**100**
Intercept	−4.13	0.07	−4.26	−4	100
Normalized wall index	0.14	0.07	0.01	0.27	**98.1**
Intercept	−4.13	0.07	−4.26	−4	100
Mean wall area	0.17	0.07	0.04	0.3	**99.4**
Intercept	−4.13	0.07	−4.26	−4	100
Mean lumen area	0.05	0.07	−0.08	0.18	77.1

Y = Calcification volume. Logit (π) = X * β, where π = P(Y = 1)^a^					
Intercept	−0.05	0.09	−0.23	0.12	72.4
Maximum wall thickness	0.69	0.1	0.49	0.89	**100**
Intercept	−0.06	0.09	−0.24	0.11	76.4
Normalized wall index	0.48	0.09	0.29	0.66	**100**
Intercept	−0.06	0.09	−0.23	0.11	74.9
Mean wall area	0.46	0.1	0.27	0.65	**100**
Intercept	−0.06	0.09	−0.23	0.11	77.1
Mean lumen area	0.04	0.09	−0.13	0.21	67.1

Y = LRNC presence (volume > 0). Log(y) = X * β + error (simple linear regressions)^1^					
Intercept	−3.11	0.05	−3.21	−3	100
Maximum wall thickness	0.39	0.05	0.29	0.5	**100**
Intercept	−3.11	0.06	−3.22	−2.99	100
Normalized wall index	0.15	0.06	0.03	0.26	**99.3**
Intercept	−3.11	0.06	−3.22	−2.99	100
Mean wall area	0.28	0.06	0.17	0.4	**100**
Intercept	−3.11	0.06	−3.22	−2.99	100
Mean lumen area	0.13	0.06	0.01	0.24	**98.6**

Y = LRNC volume. Logit (π) = X * β, where π = P(Y = 1)^b^					
Intercept	0.48	0.09	0.29	0.67	100
Maximum wall thickness	0.79	0.11	0.57	1.01	**100**
Intercept	0.42	0.09	0.24	0.59	100
Normalized wall index	0.26	0.09	0.08	0.44	**99.8**
Intercept	0.43	0.09	0.26	0.62	100
Mean wall area	0.47	0.1	0.27	0.67	**100**
Intercept	0.41	0.09	0.24	0.59	100
Mean lumen area	0.15	0.09	−0.03	0.33	95.1

*SD* standard deviations, *Prob pos eff* probability that each regression coefficient to a predictor is above 0

Interpretation of positive effect for the Bayesian simple linear regressions for calcification volumes: an increase of one standard deviation in X_j_ is expected to increase the value of Y by a multiplicative factor of exp (β_j_), all else being equal. X are predictors.

Interpretation of positive effect for the Bayesian logistic regressions for LRNC volumes: an increase of one standard deviation in X_j_ is expected to increase the odds of Y = 1 by a multiplicative factor of exp (β_j_), all else being equal. X are predictors. IPH was excluded from this analysis due to its low prevalence, limiting the robustness of statistical inference. Values in bold indicate a high posterior probability of at least 97.5% that the regression coefficient is greater than zero, corresponding to strong evidence for a positive effect.

### 3.3 Relationships between risk factors and carotid plaque characteristics

Cardiovascular risk factors demonstrated variable associations with distinct plaque features. Increasing age was associated with greater carotid calcification volume (Table 5); however, high-risk features, such as the presence of LRNC, did not increase consistently across age categories or with advancing age (Table 3, Table 5). Smoking emerged as a factor associated with a calcified plaque phenotype (Table 5).

**Table 5:** Cardiovascular risk factors for the prediction of plaque morphological data.

A. Bayesian logistic regression	Posterior mean	Posterior SD	2.50%	97.50%	Prob eff					
Y = Calcification presence (volume > 0). Logit (π) = X * β, where π = P(Y = 1)										
Intercept	−0.52	0.18	−0.87	−0.17	99.8					
Age	0.21	0.09	0.02	0.39	**98.7**					
Sex	0.17	0.2	−0.21	0.56	81.3					
Hypertension	0.77	0.2	0.38	1.16	**100**					
Diabetes mellitus	−0.05	0.33	−0.68	0.6	55.9					
Smoking	0.55	0.24	0.09	1.02	**99**					
LDL	−0.08	0.09	−0.27	0.11	80.3					
					
Y = IPH presence (volume > 0). Logit (π) = X * β, where π = P(Y = 1)										
Intercept	−3.79	0.51	−4.87	−2.86	100					
Age	0.04	0.2	−0.35	0.45	58.5					
Sex	1.14	0.52	0.18	2.23	**99.1**					
Hypertension	−0.23	0.43	−1.11	0.59	70.1					
Smoking	1	0.58	−0.19	2.08	95.3					
Diabetes mellitus	−0.8	0.61	−2.1	0.31	91.3					
LDL	0.35	0.2	−0.05	0.75	95.6					
					
Y = LRNC presence (volume > 0). Logit (π) = X * β, where π = P(Y = 1)										
Intercept	−0.17	0.18	−0.52	0.18	83.5					
Age	0.08	0.09	−0.11	0.26	79.4					
Sex	0.83	0.2	0.44	1.21	**100**					
Hypertension	−0.17	0.2	−0.57	0.22	80.6					
Diabetes mellitus	−0.01	0.33	−0.64	0.64	51.5					
Smoking	0.47	0.25	−0.01	0.96	97.4					
LDL	0.18	0.1	−0.01	0.37	96.8					
		
	*α*					*β*				
B. Bayesian ZINB model	Posterior mean	Posterior SD	2.5%	97.5%	Prob eff	Posterior mean	Posterior SD	2.5%	97.5%	Prob eff
Y = Extent of carotid calcification (no of slices). Logit (π) = X * α, where π = probability for being “at risk” (P(Y = 1)). E(y | “*at-risk*”) = r * exp(X * β), where r is an estimated dispersion parameter (not shown)										
Intercept	−0.21	0.23	−0.64	0.26	82.2	−0.04	0.26	−0.54	0.48	56.5
Age	0.2	0.11	−0.03	0.42	95.8	0.06	0.06	−0.05	0.17	85.9
Sex	0.24	0.25	−0.25	0.72	83.4	−0.03	0.12	−0.28	0.21	59.5
Hypertension	0.74	0.26	0.25	1.26	**99.8**	0.35	0.12	0.12	0.58	**99.9**
Diabetes mellitus	0.12	0.53	−0.73	1.17	57.7	−0.2	0.19	−0.58	0.16	85.9
Smoking	0.51	0.3	−0.07	1.13	95.9	0.28	0.13	0.02	0.54	**98.3**
LDL	−0.03	0.12	−0.26	0.2	60.7	−0.09	0.05	−0.2	0.02	95.2

Y = Extent of LRNC (no of slices). Logit (π) = X * α, where π = probability for being “at risk” (P(Y = 1)). E(y | “*at-risk*” = r * exp(X * β), where r is an estimated dispersion parameter (not shown)										
Intercept	−0.06	0.19	−0.43	0.32	61.9	−0.59	0.25	−1.15	−0.14	99.6
Age	0.09	0.1	−0.11	0.29	81.7	−0.03	0.04	−0.1	0.04	81.3
Sex	0.84	0.21	0.42	1.25	**100**	0.21	0.09	0.04	0.39	**99.1**
Hypertension	−0.18	0.22	−0.61	0.26	78.8	−0.03	0.08	−0.19	0.13	64.3
Diabetes mellitus	−0.09	0.35	−0.76	0.6	60.2	0.34	0.12	0.1	0.58	**99.7**
Smoking	0.49	0.27	−0.04	1.04	96.6	0.15	0.09	−0.03	0.33	95.2
LDL	0.18	0.11	−0.03	0.39	95.7	0.05	0.04	−0.02	0.13	90.1

*IPH* intraplaque hemorrhage, *LRNC* lipid-rich necrotic core, *SD* standard deviations, *LAD* left anterior descending artery, *Prob eff* probability that each regression coefficient to a predictor is above 0 for a positive posterior mean or below 0 for a negative posterior mean

A. Interpretation of an effect for the Bayesian logistic regressions for the presence of plaque morphological data: an increase of one standard deviation in X_j_ is expected to change the odds of Y = 1 by a multiplicative factor of exp (β_j_), all else being equal. X are predictors

B. Interpretation of an effect in α: an increase of one standard deviation in X_j_ is expected to change the odds of Y > 0 (the “at-risk” class) by a multiplicative factor of exp (β_j_), all else being equal. X are predictors in the Bayesian ZINB model. Interpretation of an effect in β: an increase of one standard deviation in X_j_ is expected to change E(y | “at-risk”) by a multiplicative factor of exp (β_j_), all else being equal

Values in bold indicate a high posterior probability of at least 97.5% that the regression coefficient is greater than zero, corresponding to strong evidence for a positive effect.

### 3.4 Relationships between carotid plaque characteristics and coronary artery disease

LRNC volume was not associated with the presence or extent of coronary artery disease (Table 6). However, several carotid plaque characteristics were linked to coronary findings. Increased mean wall area, calcium volume, presence of IPH, and maximum wall thickness were associated with a higher probability of nonzero CAC and greater CAC levels when CAC >0. Further, increased calcium volume and presence of IPH increased the probability of elevated coronary SIS and higher CAC when CAC >0. Notably, no significant associations were observed between carotid plaque composition and coronary plaque composition in terms of noncalcified plaques (Supplemental Table S2).

**Table 6:** Carotid plaque data for the prediction of coronary atherosclerosis.

	*α*					*β*				
	Posterior mean	Posterior SD	2.5%	97.5%	Prob eff	Posterior mean	Posterior SD	2.5%	97.5%	Prob eff
Y = CAC score. Logit (pi) = X*α, where pi = probability for being “at risk”. E(y | “*at-risk*”) = r * exp(X * β), where r is an estimated dispersion parameter (not shown)										
Intercept	1.12	0.23	0.77	1.68	100	6.56	0.13	6.32	6.82	100
Mean wall area	0.45	0.18	0.14	0.87	**99.9**	0.23	0.09	0.06	0.4	**99.7**
Intercept	1.05	0.18	0.74	1.45	100	6.59	0.13	6.33	6.82	100
LRNC volume	0	0.14	−0.24	0.3	53.1	−0.09	0.08	−0.23	0.07	85.9
Intercept	1.54	0.31	1.05	2.25	100	6.47	0.13	6.24	6.74	100
Carotid calcium volume	1.95	0.56	1.04	3.2	**100**	0.34	0.1	0.16	0.55	**100**
Intercept	1.05	0.2	0.72	1.49	100	6.56	0.14	6.3	6.83	100
IPH presence	6.17	5.76	0.23	21.02	**98.8**	0.83	0.39	0.16	1.74	**99.5**
Intercept	1.12	0.22	0.77	1.64	100	6.58	0.13	6.33	6.85	100
Maximum wall thickness	0.38	0.17	0.08	0.76	**99.5**	0.21	0.08	0.05	0.37	**99.5**
Intercept	1.1	0.2	0.76	1.56	100	6.62	0.12	6.37	6.86	100
Mean lumen area	0.28	0.15	0.01	0.61	**97.7**	0.04	0.09	−0.14	0.23	66.1

Y = Coronary SIS. Logit (pi) = X * α, where pi = probability for being “at risk”. E(y | “*at-risk*”) = r * exp(X * β), where r is an estimated dispersion parameter (not shown)										
Intercept	1.66	0.53	1.03	3.15	100	0.5	0.21	0.09	0.91	99.3
Mean wall area	0.51	0.38	0	1.48	**97.6**	0.1	0.05	0	0.2	97.2
Intercept	3.84	3.96	1.05	14.5	100	0.68	0.26	0.19	1.13	99.7
LRNC volume	2.05	3.79	−0.27	14.5	81.8	−0.01	0.05	−0.11	0.09	60.7
Intercept	2.1	0.58	1.35	3.54	100	0.42	0.17	0.07	0.76	98.9
Carotid calcium volume	2.2	0.93	0.97	4.46	**100**	0.14	0.05	0.05	0.25	**99.8**
Intercept	1.4	0.32	0.94	2.19	100	0.45	0.19	0.05	0.82	98.6
IPH presence	7.17	5.59	0.39	20.98	**99.1**	0.43	0.19	0.08	0.82	**99.2**
Intercept	2.31	2.89	1.01	12.51	100	0.51	0.24	0.07	1.03	98.9
Maximum wall thickness	0.37	0.97	−1.5	2.75	88.1	0.11	0.05	0.01	0.22	**98.2**
Intercept	1.88	0.91	1.04	4.72	100	0.58	0.23	0.12	1.02	99.2
Mean lumen area	0.56	0.52	−0.05	2.04	96.1	−0.01	0.06	−0.12	0.1	59.1

*LRNC* lipid-rich necrotic core, *IPH* intraplaque hemorrhage, *CAC* coronary artery calcium score, *SIS* Segment Involvement Score, *ZINB* zero-inflated negative binomial

The results shown are for separate models of the carotid plaque data with the corresponding carotid plaque characteristic as the only predictor in each model. Interpretation of an effect in α: an increase of one standard deviation in X_j_ is expected to change the odds of Y > 0 (the “at-risk” class) by a multiplicative factor of exp (β_j_), all else being equal. X is the carotid plaque predictor in the Bayesian ZINB model. Interpretation of an effect in β: an increase of one standard deviation in X_j_ is expected to change E(y | “at-risk”) by a multiplicative factor of exp (β_j_), all else being equal.

### 3.5 Differences between men and women

Men had a higher prevalence of coronary stenosis and coronary calcium than women. In the carotids, men had larger lumen area, greater mean wall area, and greater maximum wall thickness; these differences persisted after adjustment for body surface area (all p < 0.001). LRNC was more common in men than women (66% [242/367) vs 47% [78/166], p < 0.001), risk difference 19% points and odds ratio 2.18 (95% CI 1.50-3.17), indicating that men higher risk of LRNC than women. Sex was also associated with IPH presence, with a higher prevalence in men (Table 5). A majority of women in the study cohort were menopausal (79.5% [132/166]).

## 4 Discussion

The potential role of carotid CMR for risk stratification of carotid atherosclerosis beyond size-based measures is broadly recognized [5]. Still, the application of CMR in the study of carotid atherosclerosis has been confined to cohorts dominated by older, often symptomatic, populations with advanced carotid atherosclerosis. Expanding our understanding of CMR-detectable carotid plaque characteristics in subclinical cohorts could guide our interpretation of adverse findings from carotid CMR and other cardiovascular imaging modalities. Addressing a critical population gap in the study of carotid atherosclerosis, we present data from SCAPIS, the first study to examine a general population of asymptomatic middle-aged men and women using carotid CMR and CorCTA to assess the associations between carotid atherosclerosis, including detailed carotid plaque composition, cardiovascular risk factors, and coronary atherosclerosis.

In our population-based study of carotid atherosclerosis in middle-aged individuals, carotid plaques with calcification and LRNC are associated with different cardiovascular risk factors. This may suggest two distinct paths to carotid atherosclerosis in middle-aged individuals: one related to calcification that our data primarily links to risk factors such as age, hypertension, and smoking, and one related to the presence of LRNC that in our data is associated with elevated LDL cholesterol levels. However, calcified and lipid-rich plaques may also represent plaques at different stages of the atherosclerotic disease process. The presence of calcification, which in our study associates with coronary artery calcification, may reflect a more progressed plaque phenotype, with concomitant manifestations of atherosclerosis in other vascular beds. The presence of LRNC, on the other hand, may reflect an earlier phase of atherosclerosis, and some plaques with LRNC may progress into more stable plaque phenotypes over time.

In addition to potentially being explained by the presence of early-stage atherosclerosis in our relatively young cohort, the high prevalence of LRNC may also reflect a previously underestimated burden of higher-risk carotid plaques in contemporary middle-aged individuals. Intimal accumulation of lipoproteins occurs early during the development of atherosclerosis. Over time, lipid released by foam cells add to the growing necrotic core, a process further accelerated by ongoing inflammatory activity [21]. In the present study, 60% (320/533) of participants had at least one plaque with LRNC. Extrapolating these findings to all SCAPIS participants with carotid plaques ≥2.7 mm suggests an LRNC prevalence of around 4.3% in the middle-aged population in Sweden. The incidence of LRNC in this cohort is higher than in earlier studies. For instance, in the Multi-Ethnic Study of Atherosclerosis (MESA) cohort (data collection 2000-2002), LRNC prevalence was 48% among individuals with cardiovascular events and 18% in those without [9]. Similarly, the Atherosclerosis Risk in Communities (ARIC) study (data collection 2004-2005) reported LRNC in 44% of individuals with cardiovascular disease and 30% of those without [10]. However, with the exception of maximum wall thickness and wall area in the ARIC subcohort with cardiovascular disease, the MESA and ARIC cohorts also had larger lumen area, smaller wall area, smaller maximum wall thickness, and smaller normalized wall index, thus reflecting overall less pronounced carotid artery remodeling than in our study cohort. Nevertheless, the high prevalence of LRNC observed in the current study is concerning.

IPH is another high-risk plaque feature that is widely recognized for its strong association with both plaque progression and elevated cardiovascular risk [7], [22]. The low prevalence of IPH of ∼5% (29/533) was similar to that reported in previous studies of comparable cohorts such as participants in ARIC with 10 event-free years [10]. The prevalence of IPH was higher in men (6.8% [25/367]) than in women (2.4% [4/166]). Sex-stratified population data are scarce; most large cohorts do not stratify by sex, instead pooling sexes with adjustment or reporting composite plaque features. An exception is the Rotterdam Study, which reported higher prevalence (37% men, 27% women), likely reflecting older age (mean age ∼72 years, no upper age limit) and more progressed atherosclerosis [23]. Mechanistically, a low prevalence of IPH in individuals with early-stage atherosclerosis is plausible, as angiogenesis and associated IPH within the growing plaque likely is a later phenomenon triggered by a larger necrotic core with a hypoxic environment that stimulates neovascularization.

Several aspects of carotid atherosclerosis are associated with coronary atherosclerosis. Compared to all SCAPIS participants, all aspects of subclinical coronary atherosclerosis were more advanced in the cohort with carotid plaques [14]. Additionally, we found that several carotid plaque features, including mean wall area, carotid calcium, IPH, and maximum wall thickness, were associated with both CAC score and coronary SIS. These findings align well with a meta-analysis that associated morphological carotid plaque characteristics derived from ultrasound and CMR with CorCTA outcomes [24]. Consistent with our results, that review also did not find a link between carotid LRNC and any coronary disease score, emphasizing the urgent need for enhanced coronary plaque characterization tools capable of measuring lipid content in soft coronary plaques.

The extent of imaging-detected carotid or coronary atherosclerosis increased with age in both sexes but was consistently more prevalent in men across all age groups. However, the relative difference between men and women in the prevalence of carotid plaques was less pronounced in the CMR group compared to all SCAPIS participants [25]. Notably, women with carotid plaques exhibited a coronary atherosclerotic burden like that of men of the same age in the general population. A longitudinal study is planned to evaluate whether these women also have cardiovascular risk similar to that of men in the same age group.

## 5 Limitations

This study has several limitations. Selective participation is a common concern in population-based research aiming to describe disease prevalence. Cohort studies involving voluntary clinical examinations are often biased toward healthier individuals with higher socioeconomic status, and SCAPIS is no exception [26]. Within SCAPIS, carotid CMR was an optional, site-managed substudy. Uptake varied across sites due to logistics and scheduling, introducing potential site-level selection. However, the CMR group’s cardiovascular risk profile was broadly similar to the full ≥2.7 mm plaque group, although some risk factors and coronary findings were higher, others lower. From a broader perspective, this cohort of middle-aged individuals with asymptomatic carotid atherosclerosis represents one of the largest carotid CMR study populations to date. Differences in scanner manufacturer, coil hardware, and some sequence parameters between sites represent another limitation. Nevertheless, the vast majority (84% [510/607]) of data were obtained with the same MR scanner type with similar parameter settings and the same type of dedicated carotid MR coil. Also, while this study provided comprehensive plaque characterization, the lack of a minimum volume threshold for components such as LRNC and IPH could lead to underestimation of very small volumes. This issue is particularly relevant for IPH, whose mean volume was substantially lower than that of LRNC, as shown in Table 3, potentially reflecting a methodological challenge in accurately detecting small-scale IPH.

In conclusion, distinct cardiovascular risk factors were positively linked to the presence and extent of LRNC and calcified plaques in middle-aged individuals, suggesting diverse pathways in carotid atherosclerosis. The substantial prevalence of high-risk plaque features, particularly LRNC, highlights a significant subclinical carotid disease burden.

## Funding

This work was supported by the 10.13039/501100003793Swedish Heart-Lung Foundation (grant number 20240439), 10.13039/501100003792Swedish Brain Foundation (grant number FO2024-0069); the Henry and Ella Margareta Ståhl Foundation (grant numbers RÖ-931643, RÖ-968680, RÖ-981769, RÖ-995322); ALF Grants and Region Östergötland (grant numbers RÖ-935882, RÖ-975323). The Swedish SCAPIS trial was mainly funded by the 10.13039/501100003793Swedish Heart-Lung Foundation (grant numbers 20220129, 20240402) and had considerable support from Knut and Alice Wallenbergs Foundation, Vinnova, the Swedish Research Council and the participating Universities (Uppsala, Umea, Linkoping, Lund, Gothenburg and Karolinska Institute, Stockholm) and University Hospitals (Uppsala, Umea, Linkoping, Skane, Sahlgrenska and Karolinska). SCAPIS Linkoping received additional funding from 10.13039/100010805FORSS (the Medical Research Council of Southeast Sweden). I.G. received grants from the 10.13039/501100004359Swedish Research Council; the Swedish Heart and Lung Foundation; 10.13039/501100011077Skåne University Hospital funds; ALF grants, Lund University Diabetes Center (Swedish Research Council - Strategic Research Area Exodiab grant number 2009-1039; Linnaeus grant number 349-2006-23; the 10.13039/501100001729Swedish Foundation for Strategic Research grant number IRC15-006) and the Le Ducq Foundation. E.G. received grants from WCMM-LiU.

## Author contributions

**Oscar Soto:** Writing – review & editing, Formal analysis. **Ola Hjelmgren:** Writing – review & editing, Supervision. **David Marlevi:** Writing – review & editing, Project administration. **Bertil Wegmann:** Writing – review & editing, Software, Formal analysis. **Petter Dyverfeldt:** Writing – review & editing, Writing – original draft, Supervision, Methodology, Funding acquisition, Conceptualization. **Elin Good:** Writing – review & editing, Writing – original draft, Project administration, Methodology, Funding acquisition, Formal analysis, Conceptualization. **Tamara Bianchessi:** Writing – review & editing, Software, Data curation. **Jan Engvall:** Writing – review & editing. **Isabel Gonçalves:** Writing – review & editing, Supervision, Conceptualization. **My Troung:** Writing – review & editing, Project administration. **Linda Bilos:** Writing – review & editing, Validation. **Håkan Ahlström:** Writing – review & editing, Conceptualization.

## Ethics approval and consent

SCAPIS was approved by the Regional Ethical Review Board in Umeå, Sweden (registration number 2010-228-31 M), and all participants provided written informed consent. The present add-on carotid CMR study was additionally approved by the Swedish Ethical Review Authority (registration number 2022-04459-01).

## Consent for publication

Not applicable, as no identifiable individual data are included in this manuscript.

## Declaration of competing interests

The authors declare that they have no known competing financial interests or personal relationships that could have appeared to influence the work reported in this paper.

## Data Availability

The data underlying this article were accessed from www.scapis.org. The derived data generated in this research will be shared on reasonable request to the corresponding author and to SCAPIS.

## Footnotes

Appendix A Supplementary data associated with this article can be found in the online version at doi:10.1016/j.jocmr.2026.102686.
