# Supplementary material for: Carotid plaque characteristics and their association with cardiovascular risk factors and coronary atherosclerosis in a middle-aged population
Source: J Cardiovasc Magn Reson. 2026 Jan 8;28(1):102686. doi: 10.1016/j.jocmr.2026.102686 (PMC13126483; doi:10.1016/j.jocmr.2026.102686)
Supplement: Supplementary file 1 — Supplementary material [file mmc1.docx]

#

# SUPPLEMENTAL MATERIAL

###

### Supplemental Table S1. Parameters for High-Resolution Carotid MR Protocol

| **Parameter** | **3D Time-of-Flight** | **Pre- and post-contrast T1-weighted** | **MP-RAGE** |
| --- | --- | --- | --- |
| Imaging mode | 3D | Multi-slice 2D | 3D |
| Echo time [ms] | Site 1: 5.7  Site 2: 5.1  Site 3: 3.7  Site 4: 3.1  Site 5: 4.0 | Site 1: 10  Site 2: 10  Site 3: 10  Site 4: 13  Site 5: 10 | Site 1: 6.0  Site 2: 5.5  Site 3: 2.4  Site 4: 2.6  Site 5: 5.0 |
| Flip angle [degrees] | Site 1: 20  Site 2: 20  Site 3: 25  Site 4: 20  Site 5: 40 | Site 1: 90  Site 2: 90  Site 3: 90  Site 4: 90  Site 5: 90 | Site 1: 15  Site 2: 15  Site 3: 9  Site 4: 15  Site 5: 15 |
| Repetition time [ms] | Site 1: 17  Site 2: 20  Site 3: 21  Site 4: 20  Site 5: 15 | Site 1: 800  Site 2: 800  Site 3: 750  Site 4: 800  Site 5: 800 | Site 1: 9.7  Site 2: 9.1  Site 3:1900  Site 4: 800  Site 5: 8.4 |
| Slice thickness [mm] | Site 1: 2  Site 2: 2  Site 3: 1  Site 4: 0.8  Site 5: 2 | Site 1: 2  Site 2: 3  Site 3: 2  Site 4: 2  Site 5: 3 | Site 1: 2  Site 2: 2  Site 3: 1  Site 4: 2  Site 5: 2 |
| Pixel size [mm] | Site 1: 0.27  Site 2: 0.27  Site 3: 0.25  Site 4: 0.31  Site 5: 0.63 | Site 1: 0.50  Site 2: 0.29  Site 3: 0.25  Site 4: 0.52  Site 5: 0.35 | Site 1: 0.27  Site 2: 0.27  Site 3: 0.90  Site 4: 0.63  Site 5: 0.44 |
| Slab thickness [mm] | Site 1: 70  Site 2: 70  Site 3: 74  Site 4: 95  Site 5: 62 | Site 1: 36  Site 2: 51  Site 3: 36  Site 4: 36  Site 5: 51 | Site 1: 102  Site 2: 102  Site 3: 80  Site 4: 104  Site 5: 86 |
| Field-of-view [mm] | Site 1: 160  Site 2: 140  Site 3: 120  Site 4: 200  Site 5: 160 | Site 1: 160  Site 2: 160  Site 3: 160  Site 4: 200  Site 5: 140 | Site 1: 140  Site 2: 140  Site 3: 180  Site 4: 200  Site 5: 140 |
| Echo train length [-] | - | Site 1: 10  Site 2: 10  Site 3: 10  Site 4: 9  Site 5: 10 | Site 1: 32  Site 2: 32  Site 3: 1  Site 4: 1  Site 5: 32 |
| Contrast agent and dose [mmol/kg] |  | Site 1: Gadovist** 0.2  Site 2: Dotarem***, 0.2  Site 3: Dotarem***, 0.2  Site 4: Gadovist***, 0.2  Site 5: Dotarem***, 0.2 |  |
| * MP-RAGE images from site 3 were non-evaluable for IPH; identification of IPH therefore relied on the pre-contrast T1w images for site 3.  ** Gadovist, Bayer Schering Pharma AG, Berlin, Germany  *** Dotarem, Guerbet, Aulnay-sous-Bois, France | | | |

### Supplemental Table S2. Carotid plaque data for the prediction of coronary noncalcified plaque

| **Bayesian logistic regression** | **Posterior mean** | **Posterior SD** | **2.50%** | **97.50%** | **Prob eff** |
| --- | --- | --- | --- | --- | --- |
| Y = Noncalcified coronary plaque (volume >0). Logit (π) = X*β, where π = P(Y=1) | | | | | |
| Intercept | -1.64 | 0.12 | -1.88 | -1.42 | 100 |
| Mean Wall Area | 0.07 | 0.11 | -0.16 | 0.29 | 72.8 |
| Intercept | -1.65 | 0.12 | -1.88 | -1.42 | 100 |
| LRNC volume | 0.19 | 0.1 | -0.01 | 0.39 | 96.5 |
| Intercept | -1.64 | 0.12 | -1.87 | -1.42 | 100 |
| Carotid calcium volume | -0.03 | 0.12 | -0.28 | 0.2 | 59.1 |
| Intercept | -1.65 | 0.12 | -1.89 | -1.42 | 100 |
| IPH presence | 0.23 | 0.48 | -0.78 | 1.12 | 69.5 |
| Intercept | -1.65 | 0.12 | -1.88 | -1.42 | 100 |
| Maximum Wall Thickness | 0.14 | 0.11 | -0.09 | 0.36 | 89.3 |
| Intercept | -1.64 | 0.12 | -1.87 | -1.42 | 100 |
| Mean Lumen Area | 0.07 | 0.11 | -0.16 | 0.29 | 73 |

Prob eff = probability that each regression coefficient to a predictor is above 0 for a positive posterior mean or below 0 for a negative posterior mean.

Interpretation of an effect for the Bayesian logistic regressions for the presence of plaque morphological data: an increase of one standard deviation in X_j_ is expected to change the odds of Y=1 by a multiplicative factor of exp (β_j_), all else being equal. X are predictors. In this case there were no strong associations between carotid plaque features and coronary noncalcified plaque components.

**
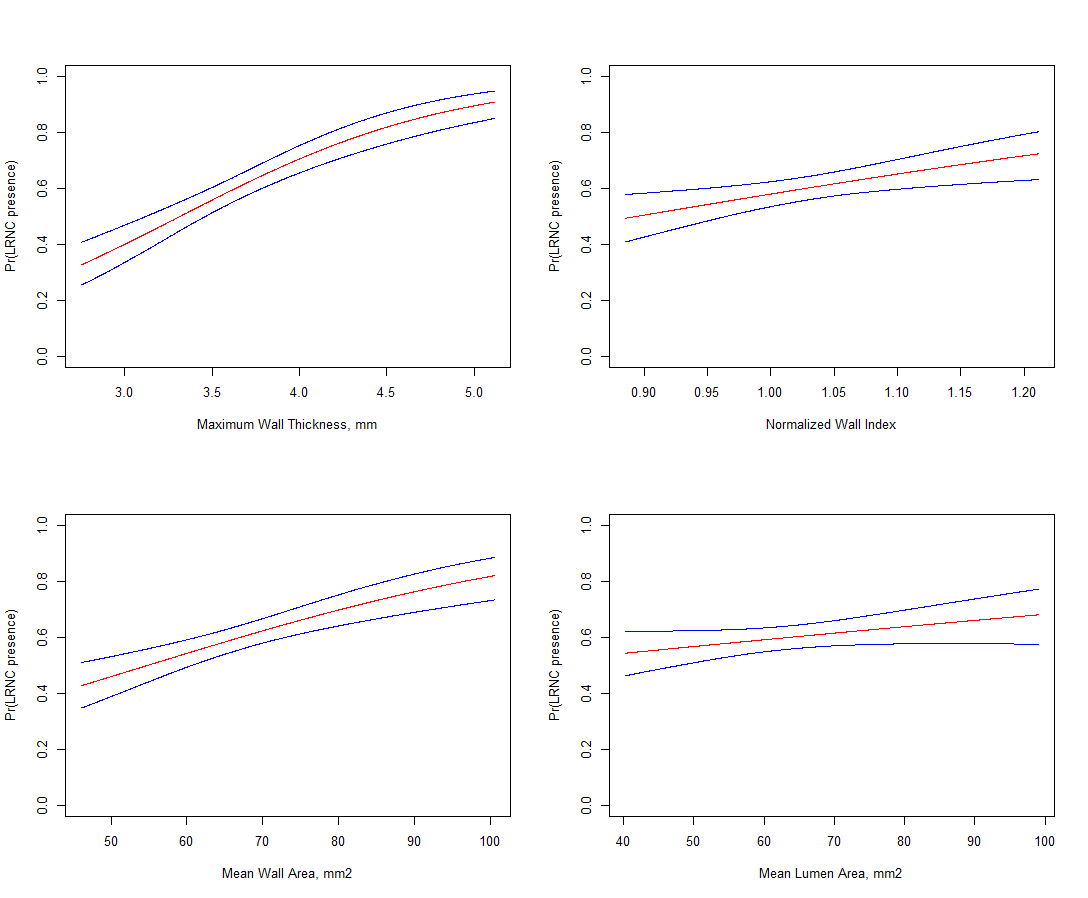
**

**Supplemental Figure S1.**  The association between morphological variables and carotid plaque LRNC. Each subplot shows the posterior probability that LRNC is present as a function of the values for each carotid plaque characteristic, where the red curve is the posterior mean and the blue curves 95 % posterior intervals for this probability.

###
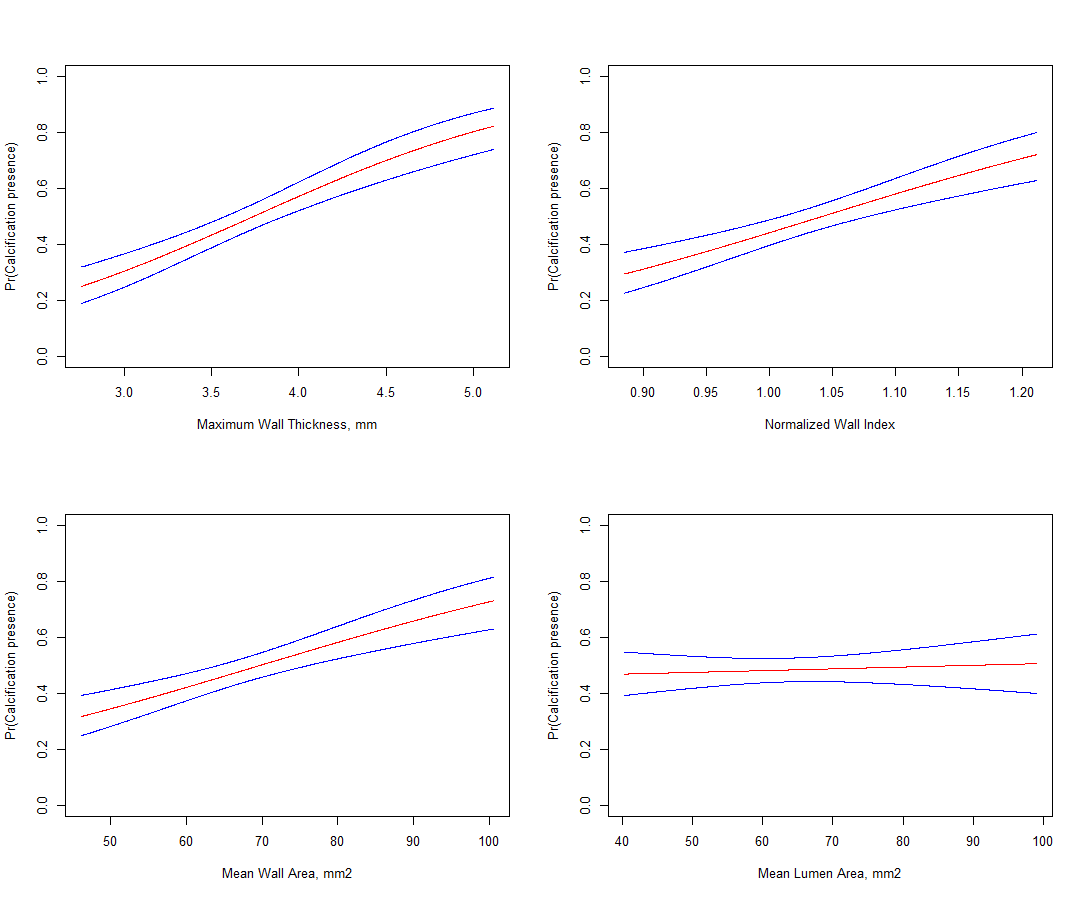


### Supplemental Figure S2. The association between morphological variables and carotid plaque calcification. Each subplot shows the posterior probability that calcification is present as a function of the values for each carotid plaque characteristic, where the red curve is the posterior mean and the blue curves 95 % posterior intervals for this probability.
